# Exploring the Universe of Natural Products: Recent Advances in Synthesis, Isolation and Structural Elucidation

**DOI:** 10.3390/plants10112368

**Published:** 2021-11-03

**Authors:** Federica Pollastro, Alberto Minassi

**Affiliations:** 1 Department of Pharmaceutical Sciences, University of Piemonte Orientale, 28100 Novara, Italy; federica.pollastro@uniupo.it; 2 PlantaChem srls, Via Canobio 4/6, 28100 Novara, Italy

Historically, plants have represented an invaluable source of compounds with complex structures and interesting pharmacological profiles. Plants’ bioactivity has been used to alleviate and treat different types of diseases, and the success of these ancient therapies gave birth to the so-called traditional medicines that have been used for thousands of years [1]. The development of the modern medicine has demonstrated even further how plants are an extraordinary source of bioactive molecules that played and still play a fundamental role in drug discovery, especially in the fight against cancer and infectious diseases [2,3].

From the discovery of penicillin through the treatment of tuberculosis with streptomycin and until the end of the nineteen sixties, natural compounds experienced a real golden age [4]. After a period of decline, where pharmaceutical industries have turned their gaze elsewhere, recently, a new interest in natural products is being felt thanks to the 2015 Nobel Prize in Medicine being awarded to Campbell and Omura for the discovery of avermectins [5] and to Youyou Tu for the discovery of artemisinin [6].

Despite this revival in interest, in the last ten years, it has been seen that the number of “new structurally unique compounds represent[s] a decreasing percentage of the total number of compounds isolated from natural sources” [7]. Given the fact that new chemical scaffolds are linked with new bioactivities, to support a possible second golden age, we cannot use traditional methods, but we need to rethink our approach to better explore still untouched areas of the plant kingdom that are retaining their “treasures”. In this way, we need a new interdisciplinary approach where analytical chemistry and synthetic chemistry work shoulder to shoulder with genome mining and synthetic biology (Figure 1).

The classical method based on the biological screening of the crude extracts is a laborious process with several limitations, and it was good for easily accessible discoveries [8,9,10,11,12]. On the other hand, a direct identification of the compounds from crude extracts by using analytical techniques could make the process more efficient, opening the door to less accessible natural products [13]. In particular, the use of metabolomics could provide accurate information on the metabolic composition of the extracts, enabling the simultaneous analysis of multiple metabolites in biological samples [14]. This approach favors the identification of new molecules, speeding up their isolation [15]. The coupling of NMR with high-resolution mass spectroscopy (HRMS) could provide fundamental indications on the structure of the main components of the plant material [16], while the high sensitivity of mass spectroscopy, coupled with databases with taxonomic information, improves the efficiency in the structure elucidation. Furthermore, the implementation of toolboxes such as the “Global Natural Products Social” (GNPS) [17] could speed up the de-replication of secondary metabolites savings time in their identification [18].

In parallel, synthetic chemistry, by using new biomimetic approaches [19,20], can furnish a laboratory alternative to obtaining the target compounds. This will be important for the complete characterization of their pharmacological profiles, and to explore their chemical space through the synthesis of analogues obtained by pinpoint modifications [21]. Despite its potentialities, synthetic chemistry suffers from several limitations just because complex architectures cannot be obtained in a flask in reasonable yields. To overcome these limitations, genome mining coupled with synthetic biology could help.

By changing the paradigm, the identification of new biogenetic pathways can lead the identification of unknown secondary metabolites [8]. Genes responsible for the synthesis of natural products are often grouped in gene clusters containing the complete genetic information required for the biosynthesis. In this field, genome mining is a powerful method to discover unknown biosynthetic pathways through the identification of the related gene clusters, opening the door to the possibility to predict the chemical structures of the bioactive natural products [22]. In parallel to genome mining, synthetic biology can redesign organisms with the insertion of specific stretches of DNA generating new biomolecular components, networks and pathway devoted to the synthesis of a promising secondary metabolite in high yields [23].

In conclusion, we are certainly facing a second golden age for natural products whose “treasures” are, however, better hidden. This requires a change in strategy with a greater integration between different analytical, biological, and chemical techniques. Although the challenge is certainly more probative, we have new and more powerful tools to be successful: we believe that the scientific progress, supported by technological advances, provides a solid basis for the discovery of new natural bioactive compounds.

## Figures and Tables

**Figure 1:**
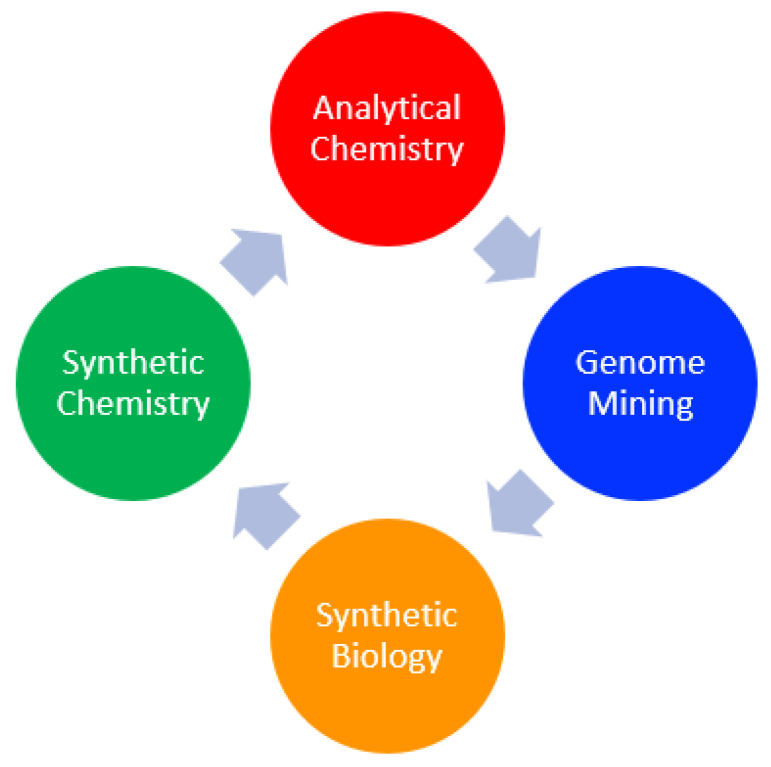
New multidisciplinary approach for the discovery of new and unique natural products.

## References

[1] Lemonnier N., Zhou G.-B., Prasher B., Mukerji M., Chen Z., Brahmachari S.K., Noble D., Auffray C., Sagner M. (2017). Traditional Knowledge-based Medicine: A Review of History, Principles, and Relevance in the Present Context of P4 Systems Medicine. Prog. Prev. Med..

[2] Cragg G.M., Newman D.J. (2013). Natural products: A continuing source of novel drug leads. BBA Gen. Subj..

[3] Thomford N.E., Senthebane D.A., Rowe A., Munro D., Seele P., Id A.M., Dzobo K. (2018). Natural Products for Drug Discovery in the 21st Century: Innovations for Novel Drug Discovery. Int. J. Mol. Sci..

[4] Katz L., Baltz R.H. (2016). Natural product discovery: Past, present, and future. J. Ind. Microbiol. Biotechnol..

[5] Campbell W.C. (2012). History of Avermectin and Ivermectin, with Notes on the History of Other Macrocyclic Lactone Antiparasitic Agents. Curr. Pharm. Biotechnol..

[6] Su X.Z., Miller L.H. (2015). The discovery of artemisinin and the Nobel Prize in Physiology or Medicine. Sci. China Life Sci..

[7] Pye C.R., Bertin M.J., Lokey R.S., Gerwick W.H., Linington R.G. (2017). Retrospective analysis of natural products provides insights for future discovery trends. Proc. Natl. Acad. Sci. USA.

[8] Tong Y., Deng Z. (2020). An aurora of natural products-based drug discovery is coming. Synth. Syst. Biotechnol..

[9] Sirignano C., Nadembega P., Poli F., Romano B., Lucariello G., Rigano D., Taglialatela-Scafati O. (2021). Triterpenoids from vitellaria paradoxa stem barks reduce nitrite levels in lps-stimulated macrophages. Plants.

[10] Leporini M., Bonesi M., Loizzo M.R., Passalacqua N.G., Tundis R. (2020). The essential oil of salvia rosmarinus spenn. From Italy as a source of health-promoting compounds: Chemical profile and antioxidant and cholinesterase inhibitory activity. Plants.

[11] Akkol E.K., Ilhan M., Kozan E., Dereli F.T.G., Sak M., Sobarzo-Sánchez E. (2020). Insecticidal activity of hyoscyamus niger l. On lucilia sericata causing myiasis. Plants.

[12] Gawlik-Dziki U., Dziki L., Anisiewicz J., Habza-Kowalska E., Sikora M., Dziki D. (2020). Leaves of white beetroot as a new source of antioxidant and anti-inflammatory compounds. Plants.

[13] Allard P.M., Genta-Jouve G., Wolfender J.L. (2017). Deep metabolome annotation in natural products research: Towards a virtuous cycle in metabolite identification. Curr. Opin. Chem. Biol..

[14] Peguero G., Gargallo-Garriga A., Maspons J., Klem K., Urban O., Sardans J., Peñuelas J. (2021). Metabolome-wide, phylogenetically controlled comparison indicates higher phenolic diversity in tropical tree species. Plants.

[15] Hubert J., Nuzillard J.M., Renault J.H. (2017). Dereplication strategies in natural product research: How many tools and methodologies behind the same concept?. Phytochem. Rev..

[16] Orbán-Németh Z., Beveridge R., Hollenstein D.M., Rampler E., Stranzl T., Hudecz O., Doblmann J., Schlögelhofer P., Mechtler K. (2018). Structural prediction of protein models using distance restraints derived from cross-linking mass spectrometry data. Nat. Protoc..

[17] Wang M., Carver J.J., Phelan V.V., Sanchez L.M., Garg N., Peng Y., Nguyen D.D., Watrous J., Kapono C.A., Luzzatto-Knaan T. (2016). Sharing and community curation of mass spectrometry data with Global Natural Products Social Molecular Networking. Nat. Biotechnol..

[18] Yang J.Y., Sanchez L.M., Rath C.M., Liu X., Boudreau P.D., Bruns N., Glukhov E., Wodtke A., De Felicio R., Fenner A. (2013). Molecular networking as a dereplication strategy. J. Nat. Prod..

[19] Caprioglio D., Salamone S., Pollastro F., Minassi A. (2021). Biomimetic approaches to the synthesis of natural disesquiterpenoids: An update. Plants.

[20] Shurpik D.N., Akhmedov A.A., Cragg P.J., Plemenkov V.V., Stoikov I.I. (2020). Progress in the Chemistry of Macrocyclic Meroterpenoids. Plants.

[21] Reymond J.L., Van Deursen R., Blum L.C., Ruddigkeit L. (2010). Chemical space as a source for new drugs. Medchemcomm.

[22] Albarano L., Esposito R., Ruocco N., Costantini M. (2020). Genome mining as new challenge in natural products discovery. Mar. Drugs.

[23] Kersten R.D., Weng J.K. (2018). Gene-guided discovery and engineering of branched cyclic peptides in plants. Proc. Natl. Acad. Sci. USA.

